# FORTIFYING THE FUTURE OF GERONTOLOGY AND GERIATRICS IN HIGHER ED: PART II—CASE STUDIES

**DOI:** 10.1093/geroni/igae098.1616

**Published:** 2024-12-31

**Authors:** Nasreen Sadeq

**Affiliations:** Chair

## Abstract

Gerontology academic programs continue to face challenges across the university setting, from low student enrollment to reduced funding and budget cuts. However, some gerontology departments are making significant changes to ensure the survival of their programs. This symposium highlights efforts to secure gerontology’s presence in higher education, including new enrollment strategies, curriculum and program changes, and interdisciplinary collaborations. First, Nasreen Sadeq and colleagues will share a case study from the University of South Florida School of Aging Studies on their efforts to increase undergraduate enrollment by developing a new Health Care Administration program. Second, Edward Miller and colleagues will describe hiring and marketing strategies that will promote growth and visibility of the Gerontology department at the University of Massachusetts Boston, such as renaming the Aging Studies major and implementing gerontology courses into the general education curriculum. Third, John Schumacher and colleagues at the University of Maryland, Baltimore County and Baltimore will share new developments in their department, including a new online Gerontology master’s program and new international research programs, and address current challenges faced by the doctoral program. Fourth, Tina Newsham and Elizabeth Fugate-Whitlock from the University of North Carolina Wilmington will describe interdisciplinary campus and community partnerships that situate the gerontology program in the university’s infrastructure, facilitate recruitment, and create meaningful opportunities for experiential learning. Finally, Robert Maiden and Danielle Gagne from Alfred University will discuss strategies for increasing enrollment, including emphasizing the interdisciplinary nature of their program and the addition of an aging concentration.

